# Intracranial Vertebrobasilar Artery Dissection Associated with Postpartum Angiopathy

**DOI:** 10.4061/2010/320627

**Published:** 2009-09-01

**Authors:** James S. McKinney, Steven R. Messé, Bryan A. Pukenas, Sudhakar R. Satti, John B. Weigele, Robert W. Hurst, Joshua M. Levine, Scott E. Kasner, Lauren H. Sansing

**Affiliations:** ^1^Department of Neurology, The Hospital of the University of PA, 3400 Spruce Street, Philadelphia, PA, 19104, USA; ^2^Department of Neurology, Robert Wood Johnson Medical School, University of Medicine & Dentistry of New Jersey, 97 Patterson Street, New Brunswick, NJ 08901, USA; ^3^Department of Neuroradiology, The Hospital of the University of Pennsylvania, 3400 Spruce Street, Philadelphia, PA, 19104, USA; ^4^Department of Neurosurgery, The Hospital of the University of Pennsylvania, 3400 Spruce Street, Philadelphia, Pennsylvania, 19104, USA; ^5^Department of Anesthesiology and Critical Care, The Hospital of the University of Pennsylvania, 3400 Spruce Street, Philadelphia, Pennsylvania, 19104, USA

## Abstract

*Background*. Cervicocephalic arterial dissection (CCAD) is rare in the postpartum period. To our knowledge this is the first reported case of postpartum angiopathy (PPA) presenting with ischemic stroke due to intracranial arterial dissection. *Case*. A 41-year-old woman presented with blurred vision, headache, and generalized seizures 5 days after delivering twins. She was treated with magnesium for eclampsia. MRI identified multiple posterior circulation infarcts. Angiography identified a complex dissection extending from both intradural vertebral arteries, through the basilar artery, and into both posterior cerebral arteries. Multiple segments of arterial dilatation and narrowing consistent with PPA were present. Xenon enhanced CT (Xe-CT) showed reduced regional cerebral blood flow that is improved with elevation in blood pressure. *Conclusion*. Intracranial vertebrobasilar dissection causing stroke is a rare complication of pregnancy. Eclampsia and PPA may play a role in its pathogenesis. Blood pressure management may be tailored using quantitative blood flow studies, such as Xe-CT.

## 1. Case Report

A 41-year-old pregnant woman with no other past medical history presented to a community hospital with pre-eclampsia at 36-week gestation. She had a systolic blood pressure (SBP) of 160 mmHg with proteinuria but no neurologic signs or symptoms. Twins were delivered by emergent Cesarean section under spinal anesthesia without apparent complications. She was discharged home on hospital day 5. 

 The following day she developed a severe headache, blurred vision, and SBP >160 mmHg. She had a generalized tonic-clonic seizure in the emergency department. She was orotracheally intubated for airway protection, treated with magnesium sulfate, levetiracetam, and labetalol. She had no further clinical seizures. A noncontrast CT of the head was normal. After extubation the following day, she became lethargic, and her mental status rapidly declined. MRI of the brain showed several areas of acute infarction in the posterior circulation. She was transferred to our neurological intensive care unit (NeuroICU) for further evaluation on the third day of hospitalization. 

**Figure 1:**
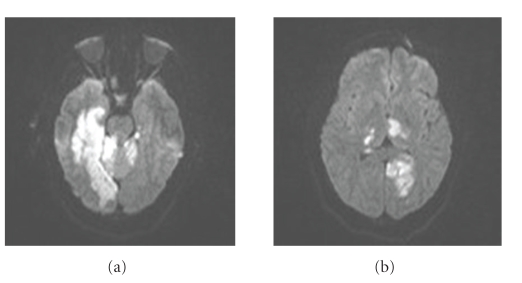
Diffusion weighted MRI demonstrates several confluent areas of restricted diffusion, consistent with infarction, confined to the bilateral posterior circulation (a,b). This was confirmed on apparent diffusion coefficient maps (not shown). No restricted diffusion was present in the anterior circulation (not shown).

 On presentation to our facility, she was lethargic and opened her eyes only to repeated tactile stimulation. She was oriented to name, could follow simple commands, and was severely dysarthric with incomprehensible speech. She had a right homonymous hemianopia, disconjugate gaze, and was quadriparetic. She was treated with a magnesium infusion, intravenous levetiracetam, aspirin 325 mg daily, and a nicardipine infusion which was titrated to maintain a goal SBP less than 120 mmHg. On the third day of hospitalization at our institution she became comatose, and her SBP was persistently >130 mmHg despite aggressive medical therapy. Continuous EEG showed diffuse slowing but no epileptiform activity. MRI of the brain redemonstrated multiple acute infarcts isolated to the posterior circulation (Figure 1). 

**Figure 2:**
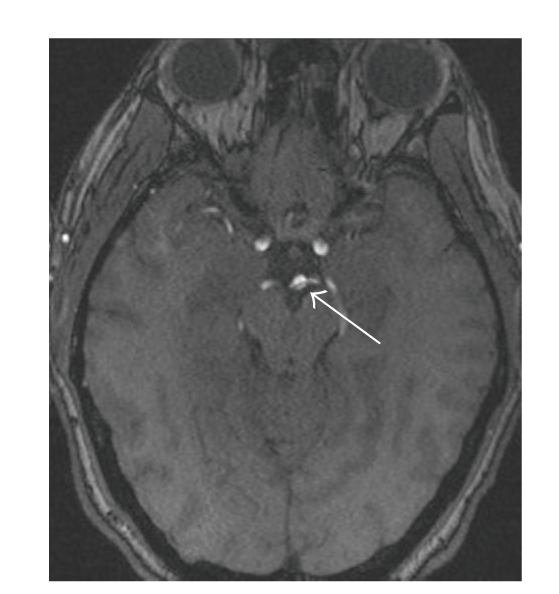
3D Time of Flight MRA source images demonstrate a linear filling defect (white arrow) in the distal basilar artery with extension into the right posterior cerebral artery (PCA) P1 segment. Additional images (not shown) demonstrated this defect also extending into the left PCA P1 segment.

There was a small area of hemorrhagic transformation of the left occipital lobe infarct seen on gradient echo images (not shown). MRA of the head and neck revealed patent cervical arteries with an apparent dissection flap visualized throughout the basilar artery (Figure 2). 

**Figure 3:**
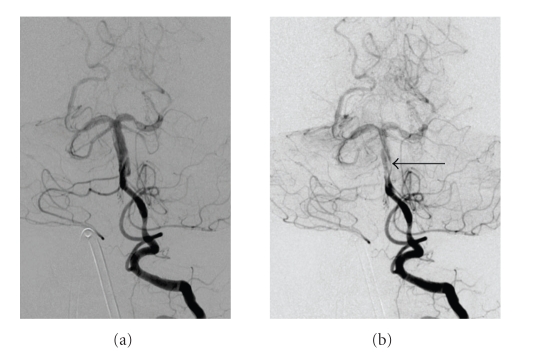
Left vertebral artery injection demonstrates poor opacification of the basilar artery and posterior cerebral arteries (PCAs) despite an adequate contrast bolus. There is marked enlargement of the distal vertebral artery in the vicinity of the vertebrobasilar junction, extending into the basilar artery and the P1 segments of both PCAs. A linear filling defect is noted originating in, and spiraling around the basilar artery and extending into the proximal bilateral PCAs, consistent with a dissection (arrow B). There are also multiple segments of vascular dilatation and narrowing in the bilateral superior cerebellar arteries and distal PCAs consistent with postpartum angiopathy.

A conventional angiogram confirmed a “double lumen” within the basilar artery with possible involvement of both intradural vertebral arteries, a finding specific for a vertebrobasilar dissection [1, 2]. In addition, distinct from the vertebrobasilar dissection, there were multiple regions of segmental narrowing and dilatation of the intracranial arteries predominantly in the posterior circulation, consistent with concomitant PPA (Figure 3). 

**Figure 4:**
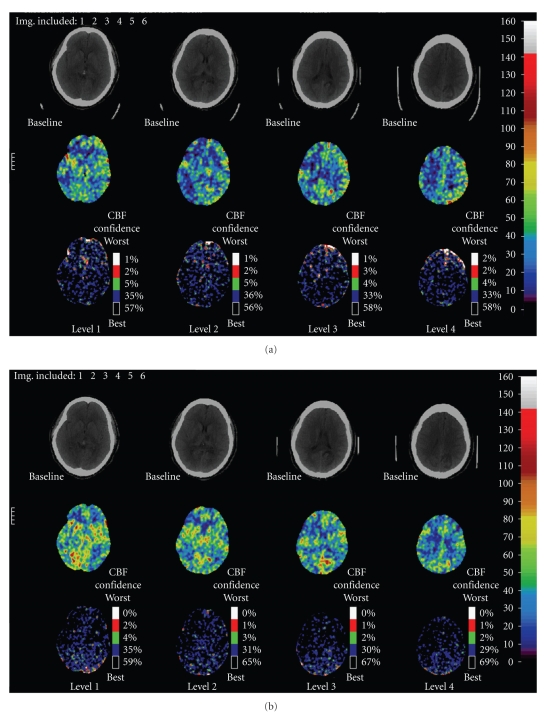
(a) Baseline Xenon perfusion study (SBP 120 mm Hg) demonstrates decreased cerebral blood flow (CBF) in both posterior cerebral artery (PCA) territories as well as in the right middle cerebral artery (MCA) and left anterior cerebral artery(ACA) territories. (b) With elevation of SBP (130 mm Hg), there is improvement in CBF in the right parietal region, but no significant improvement in the left PCA territory. There is increased perfusion in the right MCA and left ACA distributions. Increased perfusion is seen within the right occipital and left parietal lobe infarctions.

Given the radiographic findings there was uncertainty whether the patient's elevated blood pressure was contributing to her clinical deterioration or whether it was a compensatory mechanism to improve perfusion of ischemic tissue. To better direct management of her blood pressure, a Xenon enhanced CT (Xe-CT) was performed to quantify cerebral blood flow (CBF) at different blood pressures. This study showed areas of decreased relative cerebral blood volume in the bilateral posterior cerebral, right middle cerebral, and left anterior cerebral vascular territories at an SBP of 120 mmHg which improved by allowing the SBP to rise above 130 mmHg (Figure 4).

The Mg infusion was discontinued at that time, and her blood pressure target was changed to an SBP of 130 mmHg. Her neurological examination improved slowly over the next several weeks. She was discharged to an acute rehabilitation facility on hospital day 30. At the time of discharge, she was awake, alert, and oriented to person, place, and year. She had a dense right homonymous hemianopia and a moderate right hemiparesis. She required intermittent enteral feeding through a percutaneous gastrostomy tube. 

 Three months after hospital discharge, she remained in an acute rehabilitation facility. She continued to clinically improve. The gastrostomy tube had been removed, and she could walk with assistance. She continued to have significant memory difficulties and vision loss.

## 2. Discussion

Cervicocephalic arterial dissection (CCAD) is a rare postpartum complication only previously described in case reports and one recent small case series, involving a total of 6 patients [3–7]. The general annual incidence of symptomatic spontaneous CCAD is reported to be about 2.6 cases per 100000 [8]. A recent registry database identified 2.4% of cases of spontaneous CCAD occurred in the postpartum period [7]. Extrapolating this data would indicate that postpartum CCAD is as rare as 6.2 cases per 10000000 annually. In general, ischemic stroke complicating pregnancy and the postpartum period is also an uncommon condition with a reported incidence of 4.3 cases per 100000 deliveries [9]. In a series of 348295 consecutive deliveries, the most common cause of ischemic stroke was identified as eclampsia in 7 (47%) cases. The remaining etiologies were reported to be one case, each of extracranial vertebral artery dissection, PPA, amniotic fluid embolism with disseminated intravascular coagulation, and protein S deficiency. No etiology was determined in four cases. 

 The relationship between CCAD and the postpartum period remains unclear. A traumatic mechanism from strenuous labor is unlikely to be the mechanism in most cases. All 6 of the patients reported by Arnold et al. had uneventful deliveries with symptomatic dissection occurring at least 5 days postpartum, as did our patient who delivered via C-section and did not become symptomatic until day 6. A more likely association exists between CCAD and an acquired vasculopathy. In the prior series of spontaneous CCAD, 4/6 (67%) of patients who were postpartum had neuroimaging characteristics consistent with reversible cerebral vasoconstriction syndrome (RCVS), reversible posterior leukoencephalopathy (RPLS), or cortical subarachnoid hemorrhage (cSAH) without associated intradural dissection, while only 3/96 (3%) patients in the age and sex matched controls had similar findings [7]. Literature suggests that conditions such as pre-eclampsia/eclampsia, RCVS, PPA, and RPLS are all likely variable expressions of a common disease, but the underlying pathophysiology is poorly understood [10]. A common unifying theme is that surges in blood pressure and vasoactive or angiogenic substances damage the endothelial lining of vessels causing blood-brain-barrier dysfunction and accumulation of extracellular vasogenic edema. Placental growth factor (PlGF), soluble PlGF receptor (sFlt-1), and soluble endoglin are factors in the angiogenesis pathway which have been implicated in the development of PPA and eclampsia [11–13]. The resulting vessel injury causes cerebral autoregulatory dysfunction and segmental alterations in vessel tone with variable areas of arterial narrowing and dilatation [10, 14, 15]. 

 Aggressive blood pressure reduction is accepted as the treatment of hypertension in the RCVS syndromes including PPA and eclampsia [16, 17]. There are no clinical trials that have assessed treatment thresholds in these settings. There is a general consensus to lower blood pressures to less than 160/110 mmHg in patients with pre-eclampsia or eclampsia [18]. However, these recommendations are based on expert opinion and not guided by data from clinical trials. There are no clear treatment guidelines directing blood pressure management in patients with other cerebral vasoconstriction syndromes; however we routinely strive for normotensive blood pressure goals. The current American Heart Association/American Stroke Association guidelines for the management of blood pressure in acute stroke recommend permissive hypertension [19]. There seems to be no clear solution to the management of blood pressure in patients with RCVS and acute infarcts as these accepted treatments are antagonistic. In this setting, additional diagnostic studies that provide perfusion information, such as Xe-CT, CT perfusion, or MRI perfusion, can be helpful to guide management. 

 This is the first reported case of an intracranial vertebrobasilar dissection causing ischemic stroke in a patient with PPA and eclampsia. This is significant because of the uniqueness of the case and the uncertainty about blood pressure management. A flow-limiting intracranial dissection may alter the optimal target blood pressure. Blood pressure management tailored to the individual patient's cerebral hemodynamic needs is likely the best solution to optimizing CBF to areas of relative ischemia while avoiding excessive elevations in perfusion pressure which can exacerbate RCVS. Xe-CT has been utilized to determine CBF in acute stroke, differentiate between normal, ischemic, and irreversibly infarcted tissue, and optimize hemodynamic treatment parameters [20–22]. It has also previously been reported to show changes in regional CBF in a patient with eclampsia [23]. Xe-CT was used in our case to help direct our blood pressure management by presumably increasing CBF to areas of relative ischemia, while minimizing the perfusion pressure needed to accomplish this goal.

## 3. Conclusion

Intracranial vertebrobasilar dissection is a rare postpartum complication and likely occurs in vessels with endothelial damage from associated underlying conditions such as eclampsia and PPA. Antihypertensive therapy to treat these diseases may exacerbate ischemia in patients with concurrent cerebral infarction. Individualized therapy, utilizing methods to quantify CBF, may allow clinicians to optimize treatment of their patients and warrants further study.
